# P-211. Implications of Skilled Nursing Facility Origin on *Clostridioides difficile* Infection Hospitalizations in the United States

**DOI:** 10.1093/ofid/ofae631.415

**Published:** 2025-01-29

**Authors:** Kelly Reveles, Kelsey Strey, Cory T Evans, Glenn S Tillotson

**Affiliations:** The University of Texas at Austin, San Antonio, Texas; University of Texas at Austin College of Pharmacy, San Antonio, Texas; Apex PAC LLC, Apex, North Carolina; GST Micro LLC, NORTH, Virginia

## Abstract

**Background:**

*Clostridioides difficile* infection (CDI) occurs in nearly half a million Americans annually. Skilled nursing facility (SNF) residents are at increased CDI risk due to older age, underlying comorbidities, and frequent healthcare exposure. The study sought to describe the implications of CDI originating in SNFs, including CDI acuity, health outcomes, transitions of care, and economics.

**Methods:**

This was a retrospective cohort study using the Premier Healthcare Database, which represents approximately one-quarter of United States healthcare systems. Older adult (65+ years) patients were included if they had an index, non-recurrent CDI hospitalization (ICD-10 code A04.72) between July 1, 2019 and December 31, 2022. Patient and CDI characteristics, health outcomes (inpatient mortality, hospital length of stay [LOS], readmission for recurrent CDI [rCDI]), and hospital costs were compared between patients admitted from a SNF compared to a non-SNF point of origin.

**Results:**

A total of 94,330 index CDI hospitalizations were included. Patients were predominantly female (58.4%), White race (81.6%), and treated in urban settings (86.5%). Only 5.3% (n=5,003) of CDI patients were admitted from a SNF; however, 28.4% (n=26,891) were discharged to a SNF. One-third (31.0%) of patients admitted from a non-healthcare location were discharged to a SNF. Patients admitted from a SNF were older (median age 79 vs. 76 years) and more likely to have a secondary CDI diagnosis (85.0% vs. 74.4%) and emergent admission (90.2% vs. 82.1%) compared to non-SNF patients. Patients admitted from SNFs were also more likely to experience poor CDI health outcomes, including higher inpatient mortality (12.8% vs. 8.2%), readmissions for rCDI (14.8% vs. 13.8%), median hospital costs ($17,728 vs. $14,832), and longer median hospital LOS (8 vs. 7 days) (p< 0.05 for all). Median costs were significantly higher for patients discharged to a SNF compared to a non-SNF location ($19,560 vs. $11,481).

**Conclusion:**

Older, hospitalized CDI patients originating from SNFs disproportionately experience poor outcomes in health and financial burden. Over one-quarter of CDI patients were discharged to a SNF suggesting a major impact on physical function, quality of life, and associated risk.

**Disclosures:**

**Kelly Reveles, PharmD, PhD**, AstraZeneca: Grant/Research Support|Ferring Pharmaceuticals: Advisor/Consultant

